# Insight Into Pituitary lncRNA and mRNA at Two Estrous Stages in Small Tail Han Sheep With Different *FecB* Genotypes

**DOI:** 10.3389/fendo.2021.789564

**Published:** 2022-02-01

**Authors:** Si Chen, Xiaofei Guo, Xiaoyun He, Ran Di, Xiaosheng Zhang, Jinlong Zhang, Xiangyu Wang, Mingxing Chu

**Affiliations:** ^1^ Key Laboratory of Animal Genetics and Breeding and Reproduction of Ministry of Agriculture and Rural Affairs, Institute of Animal Science, Chinese Academy of Agricultural Sciences, Beijing, China; ^2^ Institute of Animal Husbandry and Veterinary Medicine, Tianjin Academy of Agricultural Sciences, Tianjin, China

**Keywords:** Small Tail Han sheep, pituitary, *FecB*, lncRNA, fecundity

## Abstract

The pituitary is a remarkably dynamic organ with roles in hormone (FSH and LH) synthesis and secretion. In animals with the *FecB* (fecundity Booroola) mutation, the pituitary experiences hormone fluctuations during the follicular–luteal transition, which is implicated in the expression and regulation of many genes and regulators. Long non-coding RNAs (lncRNAs) are a novel type of regulatory factors for the reproductive process. Nevertheless, the expression patterns of lncRNAs and their roles in *FecB-*mediated follicular development and ovulation remain obscure. Thus, we profiled the pituitary transcriptome during the follicular (F, 45 h after evacuation vaginal sponges) and luteal (L, 216 h after evacuation vaginal sponges) phases in *FecB*-mutant homozygous (BB) and wild-type (WW) Small Tail Han sheep. We identified 78 differentially expressed genes (DEGs) and 41 differentially expressed lncRNAs (DELs) between BB_F and BB_L, 32 DEGs and 26 DELs between BB_F and WW_F, 16 DEGs and 29 DELs between BB_L and WW_L, and 50 DEGs and 18 DELs between WW_F and WW_L. The results of real-time quantitative PCR (RT-qPCR) correlated well with the transcriptome data. In both the follicular and luteal phases, DEGs (*GRID2*, glutamate ionotropic receptor delta type subunit 2; *ST14*, ST14 transmembrane serine protease matriptase) were enriched in hormone synthesis, secretion, and action. MSTRG.47470 and MSTRG.101530 were the *trans*-regulated elements of *ID1* (inhibitor of DNA binding 3, HLH protein) and the DEG *ID3* (inhibitor of DNA binding 3, HLH protein), and *EEF2* (eukaryotic translation elongation factor 2), respectively; these factors might be involved in melatonin and peptide hormone secretion. In the *FecB*-mediated follicular phase, MSTRG.125392 targeted seizure-related 6 homolog like (*SEZ6L*), and MSTRG.125394 and MSTRG.83276 targeted the DEG *KCNQ3* (potassium voltage-gated channel subfamily Q member 3) in *cis*, while MSTRG.55861 targeted *FKBP4* (FKBP prolyl isomerase 4) in *trans*. In the *FecB*-mediated luteal phase, LOC105613905, MSTRG.81536, and MSTRG.150434 modulated *TGFB1*, *SMAD3*, *OXT*, respectively, in *trans*. We postulated that the *FecB* mutation in pituitary tissue elevated the expression of certain genes associated with pituitary development and hormone secretion. Furthermore, this study provides new insights into how the pituitary regulates follicular development and ovulation, illustrated by the effect of the *FecB* mutation.

## Introduction

The *FecB* (fecundity Booroola) mutation was first detected in high-fecundity Australian Booroola Merino sheep (1). Mulsant et al. (2) Souza et al. (3), and Wilson et al. (4) revealed that the effect of *FecB* mutation was due to the substitution of a base (A to G at 746 bp, A746G) in the *BMPR1B* (bone morphogenetic protein receptor 1B) coding region on ovine chromosome 6, resulting in the corresponding substitution of one amino acid (glutamine to arginine, Q249R). Through an impairment of the activity of the BMPR1B receptor kinase, *FecB* mutation weakened the inhibitory effect of the *BMPR1B* gene on steroid synthesis in granulosa cells, leading to the maturation and ovulation of ovulatory follicles at significantly small diameters and fewer granulosa cells in homozygous *FecB* sheep than those in non-*FecB* sheep (2, 3, 5–7). In our previous study, litter size was significantly correlated with the *FecB* genotype in high-prolificacy Chinese Small Tail Han sheep (8). The gonadotropic hormones follicle-stimulating hormone (FSH) and luteinizing hormone (LH) are the dominant hormones in the coordinate regulation of follicle development and antral follicle ovulation (9). A previous study predicted that *FecB* might impact the pituitary gland through the regulation of hormone release during one estrus cycle in ewes (10). At a particular physiological time during the follicular phase, the plasma concentration of FSH in *FecB* homozygous ewes was higher than that in non-*FecB* ewes (11–13). The follicular–luteal transition is a complex and elaborate process that involves pituitary hormones and several cell-specific gene products that are under strict developmental regulation. The identification of the key regulators of these developmental processes could be beneficial for understanding the mechanisms of how *FecB* regulates the follicular–luteal transition at the molecular level and for screening candidate protein-coding genes with impacts on reproductive ability in ewes.

Previous transcriptomics studies have focused solely on protein-coding genes (14). Nevertheless, transcriptomic analyses and chromatin signatures showed a plethora of non-coding transcripts produced by eukaryotic cells (15, 16). Of these, long non-coding RNAs (lncRNAs) are a heterogeneous group of non-protein-coding transcripts of greater than 200 nucleotides in length with unknown coding potential. They include circular RNAs, long intergenic RNAs, pseudogenes, and antisense RNAs (17). Accumulating evidence has implicated lncRNAs in a wide array of cellular processes, such as transcriptional regulation, cellular pluripotency and reprogramming, and differentiation (reviewed in 18, 19). In terms of transcriptional regulation, lncRNAs act in *cis* or *trans* through the recruitment of RNAs and proteins. *Cis*-acting lncRNAs activate, repress, or modulate gene expression in a manner dependent on the location of their own transcription sites (20). Unfortunately, it has been difficult to accurately identify lncRNA sequences from transcribed sequences. *Trans*-acting lncRNAs affect nuclear structure and organization and interact with and modulate the activity of RNAs and proteins (21). The cytoplasmic localization of lncRNAs and the biological activities they participate in suggest that some lncRNAs can regulate the secretion of reproductive hormones.

Pituitary gonadotropins form the center of the endocrine axis involved in the reproductive process. At puberty, hypothalamic gonadotropin-releasing hormone (GnRH) stimulates the pituitary tissue to synthesize FSH and LH. Both the FSH and LH receptors are G protein-coupled receptors and have cascade amplification effects on the estrous or menstrual cycle. Gonadotropins are produced at demanded levels and specific times in response to multiple hormonal signals; thus, regulatory elements (lncRNAs) are expected to play key roles in both the stage specificity and hormonal responsiveness of their expression. Several lncRNAs have been proposed to affect the signal transduction of FSH and LH. In rats, the pituitary intergenic lncRNA m433s1 promoted the expression level of *FSHB* and the secretion of FSH by functioning as a competing endogenous RNA of miR-433 and reducing its inhibition of *FSHB* messenger RNA (mRNA) (22). In humans, silencing the pituitary lncRNA UCA1 significantly suppressed the secretion of prolactin secretion and the growth of pituitary cancer cells (23). In ram pituitary cells, short-interfering RNA knockdown of lncRNA-TCONS_00066406 reduced the mRNA levels of *FSHB* and *LHB* (24). *FecB* mutation has been reported to cause putative transcriptional changes in the pituitary. Pituitary MSTRG.259847.2 was upregulated in *FecB* homozygous ewes compared with *FecB* heterozygous ewes. A subsequently constructed lncRNA–mRNA interaction network highlighted *Smad2*, and experiments at the cell level showed that MSTRG.259847.2 can inhibit *Smad2* and *LHB* (25). However, *FecB* mutation reduced the expression levels of some genes in the pituitary, including *BMP2*, *TGFB2*, *TGFB3*, *ID1*, and *ID3* (26). Studies identifying lncRNAs associated with the follicular–luteal transition have been performed mainly on ovarian tissues, and investigation of lncRNAs at the pituitary level in ewes with different *FecB* status and during different stages of estrus has been rarely reported.

Small Tail Han sheep are an indigenous Chinese high-fecundity sheep breed with year-round estrus and a high ovulation rate (27). The estrus cycle consists of the follicular and luteal phases, accompanied by changes in the concentrations of hormones such as FSH and LH. Our previous study observed that *FecB*-mutant ewes regulate the expression levels of the *BMPR1B*, *BMP15*, and *GDF9* genes, which are relevant to hormone and signal transduction (28)—that is, *FecB* mutation might affect the follicular–luteal transition to further influence sheep fertility. In this study, we collected pituitary tissues to analyze how the pituitary regulates follicular development, maturation, and ovulation in the context of *FecB* mutation. Our data provided a useful resource for further studying the functional roles of these genes and lncRNAs in the follicular development and ovulation of BB genotype ewes.

## Materials and Methods

### Ethical Consideration

All experimental ewes were housed and fed by the Science Research Department of the Institute of Animal Sciences, Chinese Academy of Agriculture Sciences (IAS-CAAS), and ethical approval was received from the Animal Ethics Committee of the IAS-CAAS (no. IAS 2019-49).

### Experimental Design and Pituitary Collection

Based on TaqMan genotyping (29), six *FecB*-mutant homozygous (BB, litter size = 2.2 ± 0.6) and six *FecB* wild-type (WW, litter size = 1.0 ± 0.0) Small Tail Han ewes were selected from a nucleus herd (Shandong, China). The selected ewes were approximately 3 years old and were similar in weight (**Table 1**). All non-pregnant animals had free access to food and water under natural temperature and lighting conditions. We divided genotype BB and WW ewes into a follicular phase group and a luteal phase group. Estrus synchronization was performed in September, within the usual breeding period (from August to November) described for this breed in Tianjin (117.2 E, 39.13 N). Firstly, ewes were treated with vaginal sponges (300 mg progesterone; InterAg Co., Ltd., Hamilton, New Zealand), followed by injection of vitamin AD to protect the endometrium. The vaginal sponges were evacuated after 12 days, and the removal time was set as 0 h. In accordance with previous reproductive trait analyses, six ewes (three BB and three WW ewes) were euthanized at 45 h (follicular phase) and six ewes (three BB and three WW ewes) at 216 h (luteal phase) (30, 31). Pituitary samples were dissected immediately after euthanasia, frozen in liquid nitrogen, and stored at −80°C for further analysis.

**Table 1:** Litter size and body condition characteristics of genotype BB and WW ewes.

Group	AA (years)	OR	LS	AW (kg)
BB ewes	2.8 ± 0.6	2.4 ± 0.3**	2.2 ± 0.6**	61.26 ± 5.16
WW ewes	3.0 ± 0.5	1.0 ± 0.0**	1.0 ± 0.0**	63.54 ± 7.37

BB, FecB-mutant homozygous; WW, FecB wild type; AA, average age; OR, ovulation rate; LS, litter size; AW, average weight.

**p ≤ 0.01.

### RNA Isolation, Library Construction, and Sequencing

Total RNA was extracted from the pituitary using the TRIzol reagent (Thermo Fisher Scientific, Waltham, MA, USA). The purity, concentration, and integrity of RNA were checked with a Nano Photometer^®^ spectrophotometer (IMPLEN, Westlake Village, CA, USA), Qubit^®^ RNA Assay kits (Thermo Fisher Scientific, Waltham, MA, USA), and RNA Nano 6000 Assay, respectively. The RNA integrity number (RIN) value of all the samples was greater than 7.

Three micrograms of total RNA was used as the starting amount for the construction of an lncRNA library. Ribo-Zero™ Gold Kits (Epicenter, Madison, WI, USA) were applied to remove ribosomal RNA (rRNA) from the total RNA. Following the instructions for the NEB Next^®^ Ultra Directional RNA Library Prep Kit for Illumina^®^ (NEB, Ipswich, MA, USA), 12 RNA sequencing libraries were constructed for paired-end sequencing. Pooled libraries were sequenced on the Illumina HiSeq X platform (Illumina, San Diego, USA) using a chain-specific library construction strategy and the number and structure of the mRNA, known lncRNA, and novel lncRNA transcripts. All the experimental sequencing data were generated by Annoroad Gene Technology Co., Ltd. (Beijing, China).

### Data Quality Control and Assembly

To ensure the quality of further analytical data, raw reads were filtered using in-house Perl scripts (Annoroad Gene Technology Co., Ltd, Beijing, China) to obtain high-quality reads. The filtering step involved deleting reads with low quality (Phred quality score <5%), adapter contamination, matches to rRNA sequence, or a rate of ambiguous bases higher than 5% (32). The Phred quality score refers to the rate of sequencing errors for a given base; for example, Q30 indicates that the base sequencing error rate was 0.1%. We aligned paired-end clean reads to the oar4.0 sheep reference genome (https://www.ncbi.nlm.nih.gov/assembly/GCF_000298735.2) using HISAT2 (v.2.0.5) (33) with the parameters “–rna-strandness RF” and “–dta -t -p 4”. Only the uniquely mapped reads were assembled, and the expression levels were predicted using String Tie (v.1.3.2d) (33) with the parameter “-G ref.gtf -rf -1”. Thereafter, we calculated the number and ratio of the uniquely mapped reads within the three gene functional elements: exons, introns, and intergenic elements. Homogeneity analysis was subsequently performed to guarantee that the sequencing results did not impact further transcriptome analysis. Thus, mRNA and known lncRNA transcripts were identified.

### Identification of Potential lncRNA Candidates

To decrease the false-positive rate, the screening of candidate lncRNAs is divided into two steps: one is based on the position of the coding reads [intronic lncRNA, long intergenic non-coding RNA (lincRNA), and antisense lncRNA] and the other is to count the encoding potential (34). The following were applied: 1) Transcripts with lengths longer than 200 bp, exon numbers greater than 2, and read coverage greater than 5 were retained. 2) The GffCompare program (v.0.10.1) was used to annotate the assembled transcripts and discard known mRNAs and other non-coding RNAs (rRNAs, tRNAs, snoRNAs, etc.) (35). 3) Transcripts with class_code “i” (potential intronic lncRNA), “x” (potential antisense lncRNA), “u” (potential intergenic lncRNA), “j” (potential novel isoform with more than one splice junction of a reference transcript), or “o” (genetic exonic overlapping with a reference transcript) were retained (36). 4) The Coding–Non-Coding Index (CNCI), Coding Potential Calculator (CPC), the Pfam database, and the Coding Potential Assessment Tool (CPAT) were utilized to predict the coding potential of preliminary candidate lncRNAs. The results predicted by the four aforementioned tools were intersected to obtain potential lncRNA candidates. The CNCI was determined by profiling adjoining nucleotide triplets to distinguish coding/non-coding transcripts independent of known annotations (37), with the parameter “–score 0 –length 199 –exon_num 2”. CPC can access the protein-coding potential of each transcript according to six biologically meaningful sequence features (38). Both CNCI and CPC are dependent on a score <0, thus defining lncRNA transcripts as non-coding RNAs. In Pfam, the profile hidden Markov models (HMMs) were searched for protein domains to obtain transcripts with known protein domains, based on the UniProt Knowledgebase (UniProtKB) (39). LncRNAs could be regarded as non-coding RNAs when the *E*-value ≥0.001. CPAT (v.1.2.1) works on a logistic regression model with four sequence features: Fickett TESTCODE statistic, open reading frame (ORF) size, ORF coverage, and hexamer usage bias (40).

### Differential Expression Analysis of lncRNAs and mRNAs and Clustering

To verify the rationality of the samples and the reliability of the experiment, the correlation of the expression levels between samples was examined using Pearson’s correlation methods. Gene expression levels are generally measured on the basis of the amount of mRNA transcribed from a gene. Based on the reference GTF file and the HISAT BAM files, the HTSeq Python package (v.0.6.1) was used to calculate read counts, with the parameters “-I gene_id -f bam -s” and “reverse -a 10 -q”. Because three biological replicates were performed for each experimental group, the DEseq2 package (v.1.28.1) was used for the analysis of differential expression to calculate the log_2_(fold change) and *p*-value based on the negative binomial distribution model (41). Meanwhile, log2(fold change) > 1 and *p*_adj_ < 0.05 were regarded as the essential criteria to identity the mRNAs and lncRNAs that were significantly differentially expressed in BB_F *vs*. BB_L (different estrus stages under genotype BB ewes, named gene set 1), BB_F *vs*. WW_F (different *FecB* genotypes ewes in the follicular phase, named gene set 2), BB_L *vs*. WW_L (different *FecB* genotypes ewes in the luteal phase, named gene set 3), and WW_F *vs*. WW_L (different estrus stages under genotype WW ewes, named gene set 4) individually. The intersection of BB_F *vs*. BB_L and WW_F *vs*. WW_L was named gene set 5; genes in this set were considered highly conserved during the follicular–luteal transition and to be potentially affected at the transcriptional levels by the *FecB* mutation. The intersection of BB_F *vs*. BB_L and BB_F *vs*. WW_F was named gene set 6; these genes were implicated in the process of follicular development and ovulation modulation by *FecB*. StringTie (v1.3.2d) was adopted to count the fragments per kilobase of transcripts per million mapped reads (FPKM). Subsequently, systematic clustering analysis based on the log_2_(FPKM) value of each mRNA and lncRNA was performed with pheatmap (v.1.0.2) to analyze the similarities and relationships between the different libraries (42). The analyses included Pearson’s correlation and Euclidean distance methods.

### Target Gene Prediction for Differentially Expressed lncRNAs and Network Construction

The relationships between lncRNAs and potential protein-coding genes were predicted on the basis of their distances and expression correlations, including the *cis*- and *trans*-acting relationships. Protein-coding genes adjacent to the lncRNAs (100 kb upstream and downstream) were identified as potential *cis*-target genes (43). Potential *trans*-target genes were predicted using Spearman’s correlation coefficients between the expressions of mRNAs and lncRNAs (*r* ^2^ ≥ 0.95) (43). According to the *cis*- or *trans*-acting relationship between the differentially expressed lncRNAs (DELs) and target genes, we visualized the lncRNA–mRNA networks with Cytoscape (v.3.8.0), which was run with layout = “attribute circle layout”.

### Functional Annotation of Candidate Genes

To reveal the potential roles of DELs, we performed Gene Ontology (GO) category and Kyoto Encyclopedia of Genes and Genomes (KEGG) pathway enrichment analyses on their target genes using the clusterProfiler package (v.3.16.0) with the false discovery rate (FDR) pAdjustMethod. GO analysis (http://geneontology.org) provides structured and quantifiable knowledge concerning the functions of genes and gene products, and the molecular function ontology represents the activity of the gene products, especially focusing on transcription regulator activities (44). KEGG systematically analyzes gene functions, linking genomic information with functional information (45). Furthermore, KEGG analysis can predict the protein interaction networks involved in various cellular processes. A GO term or a KEGG pathway with corrected *p* < 0.05 was considered significantly enriched. We mainly focused on the pathways related to reproduction and pituitary function.

### RT-qPCR Validation

Six mRNAs and six lncRNAs were randomly selected for validation *via* real-time quantitative PCR (RT-qPCR). *β-actin* was used as a control. The primers were designed with Primer Premier 5 and synthesized by Qine Ke Biotech (Beijing, China) (**Supplementary Table S6**). For RT-qPCR analysis, 1 μg of total RNA was reverse transcribed to complementary DNA (cDNA) following the instructions in the PrimeScript™ RT reagent Kit with gDNA Eraser (Takara, Beijing, China). The genes and lncRNAs were diluted fourfold before use for RT-qPCR according to the instructions in TB Green^®^ Premix Ex Taq™ II (Takara, Beijing, China) and analyzed with QuantStudio^®^ 3 (ABI, Foster City, CA, USA). All genes and lncRNAs were amplified in triplicate. The data of each gene and lncRNA were analyzed with the 2^−ΔΔCt^ method (46). The correlation between the RT-qPCR data and the FKPM value from the RNA sequencing (RNA-seq) data was calculated using Pearson’s correlation method in R (v.4.0.2).

## Results

### Overview of the Sequencing Data in Small Tail Han Sheep Pituitary Tissue

The raw RNA-seq data from the Small Tail Han sheep were analyzed for quality control before aligning reads to the reference genome. More than 97.50 Gb of clean read sequences were generated from each library, with a corresponding rate of clean Q30 bases higher than 91.24%, indicating that the filtered sequence was appropriate for mapping. All the high-quality clean reads were mapped to the oar4.0 sheep reference genome using HISAT2. The percentage of the total mapped and uniquely mapped reads in these samples reached 92.66% and 88.27%, respectively. The rate of multi-mapping reads from the 12 libraries was less than 4.62% (**Supplementary Table S1**).

### Identification of lncRNAs and mRNAs in Small Tail Han Sheep Pituitary Tissue

**Figure 1:**
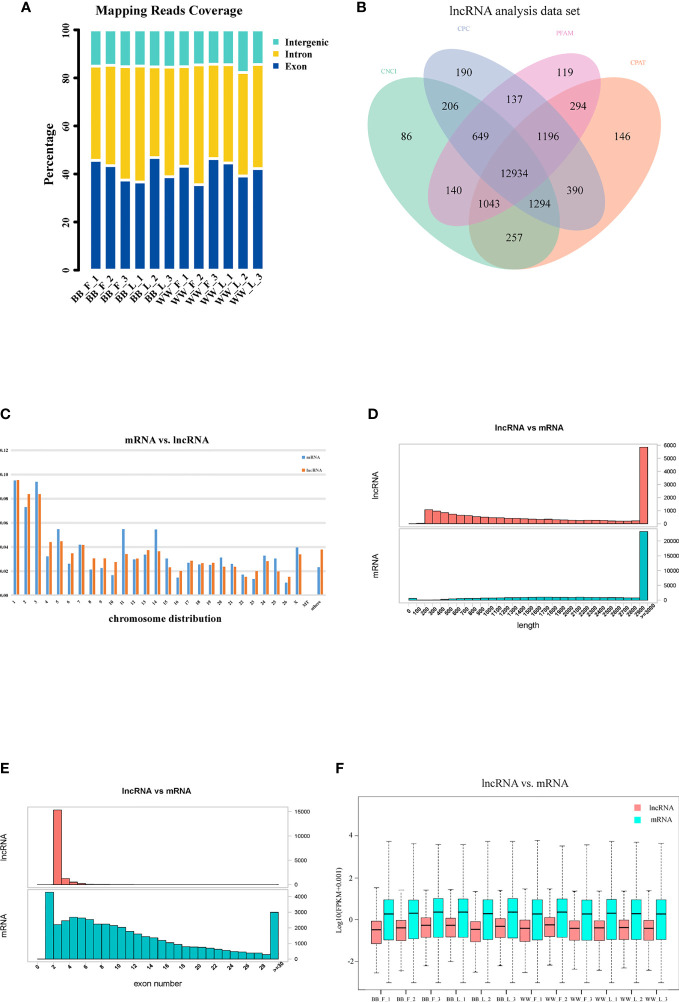
Identification of long non-coding RNAs (lncRNAs) and messenger RNAs (mRNAs) in Small Tail Han sheep pituitary. **(A)** Classification of the uniquely mapped read locations, including exon, intron, and intergenic regions. **(B)** Venn diagram analysis showing the number of common and unique novel lncRNAs identified by four methods: Coding–Non-Coding Index (CNCI), Coding Potential Calculator (CPC), the Pfam database, and the Coding Potential Assessment Tool (CPAT). **(C)** Distribution of lncRNAs and mRNAs on chromosomes. **(D)** Lengths of lncRNAs and mRNAs. **(E)** Exon numbers of lncRNAs and mRNAs. **(F)** FPKM value of lncRNAs and mRNAs.

According to their locations, the unique reads from 12 samples were categorized into three types: intergenic, intronic, and exon (**Figure 1A**). Approximately 15.17% of the lncRNAs were distributed in intergenic regions, 42.85% were introns overlapping lncRNAs, and 41.98% were exons overlapping lncRNAs. After screening preliminary candidate lncRNAs, the intersecting results of the CNCI, CPC, Pfam, and CPAT tools were used to obtain potential lncRNA candidates, shown in **Figure 1B**. Furthermore, we identified the essential features of the lncRNAs and contrasted them with those of mRNAs. The genome distribution of most mRNAs and lncRNAs was randomly assigned to the 26 autosomes and the X chromosome; 480 mRNAs (2.3%) and 431 lncRNAs (3.8%) not aligned with any chromosomal location, and 10 mRNAs and 1 lncRNA were found to align to a mitochondrial location (**Figure 1C**). The length distribution of the lncRNAs was mainly appropriately 2,900–3,000 bp, similar to that of the mRNA transcripts in pituitary tissue (**Figure 1D**). Most lncRNAs possessed two exons, significantly fewer than the average number of mRNA transcripts (**Figure 1E**). The expression abundance of lncRNAs was lower than that of mRNAs in the 12 samples (**Figure 1F**).

### Profiling of DE lncRNA and DEGs in Small Tail Han Sheep Pituitary

**Figure 2:**
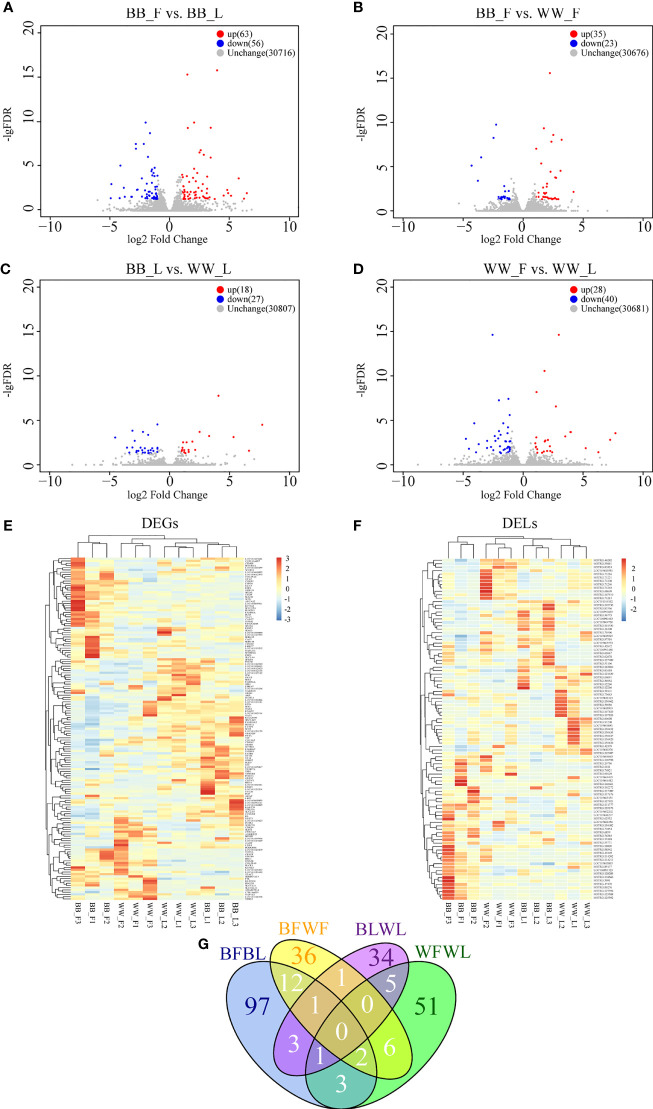
Analysis of differentially expressed lncRNAs (DELs) and differentially expressed genes (DEGs). **(A–D)** Volcano plots showing the upregulated and downregulated genes in BB_F *vs*. BB_L **(A)**, in BB_F *vs*. WW_F **(B)**, in BB_L *vs*. WW_L **(C)**, and in WW_F *vs*. WW_L **(D)**. **(E)** Hierarchical clusters of DEGs, where all the fragments per kilobase of transcripts per million mapped reads (FPKM) values of genes were normalized by log_10_(FPKM). **(F)** Hierarchical clusters of DELs, where all the FPKM values of known and novel lncRNAs were normalized by log_10_(FPKM). **(E)** Venn diagram showing the analysis of unique and shared DEGs and DELs between the BB and WW groups and between the follicular and luteal groups. **(G)** Venn diagram showing the analysis of unique and shared DEGs and DELs between the BB and WW groups and between the follicular and luteal groups. *F*, follicular phase; *L*, luteal phase, *BB*, *FecB*-mutant homozygous genotype; *WW*, *FecB* wild-type genotype.

The correlation coefficient of the gene expression levels between samples was higher than 0.80, indicating that the sample selection was consistent and reliable. Under the criteria of log2(fold change) > 1 and *p*_adj_ < 0.05, 78 differentially expressed genes (DEGs; 37 upregulated and 41 downregulated) and 41 DELs (26 upregulated and 15 downregulated) were screened between BB_F and BB_L (**Figure 2A** and **Supplementary Table S2**), 32 DEGs (20 upregulated and 12 downregulated) and 26 DELs (15 upregulated and 11 downregulated) were screened between BB_F and WW_F (**Figure 2B** and **Supplementary Table S2**), 16 DEGs (9 upregulated and 7 downregulated) and 29 DELs (9 upregulated and 20 downregulated) were screened between BB_L and WW_L (**Figure 2C** and **Supplementary Table S2**), and 50 DEGs (19 upregulated and 31 downregulated) and 18 DELs (9 upregulated and 9 downregulated) were screened between WW_F and WW_L (**Figure 2D** and **Supplementary Table S2**). The mRNA and lncRNA expression patterns were similar in both the follicular and luteal phases. Nevertheless, a different expression profile was observed at the follicular–luteal transition, which may indicate that changes in transcript expression accompanied *FecB* mutation (**Figures 2E, F** **)**. Furthermore, we compared the quantity and distribution of the DEGs and DELs in each of the four pairwise comparisons. The DEGs and DELs from the intersection of BB_F *vs*. BB_L and WW_F *vs*. WW_L were involved in follicular–luteal transition and conserved across the different genotypes, including *GRID2*, LOC105610376, MSTRG.163804, and MSTRG.81824. The intersection of BB_F *vs*. BB_L and BB_F *vs*. WW_F contained vital genes and lncRNAs, including *LRRC7*, *INSM2*, *SEZ6L*, *BMX*, *KCNQ3*, *GRM8*, *LDB3*, *CDH12*, LOC101109936, MSTRG.125394, MSTRG.125388, MSTRG.125392, MSTRG.83276, and MSTRG.81824, which are involved in the process by which *FecB* mutations affect follicular development. The intersection of BB_F *vs*. BB_L and BB_L *vs*. WW_L included LOC105610376, MSTRG.26398, MSTRG.127921, and MSTRG.134082, which are involved in ovulation in *FecB* mutant ewes (**Figure 2G**).

### GO and Pathway Enrichment Analysis of DEGs

**Figure 3:**
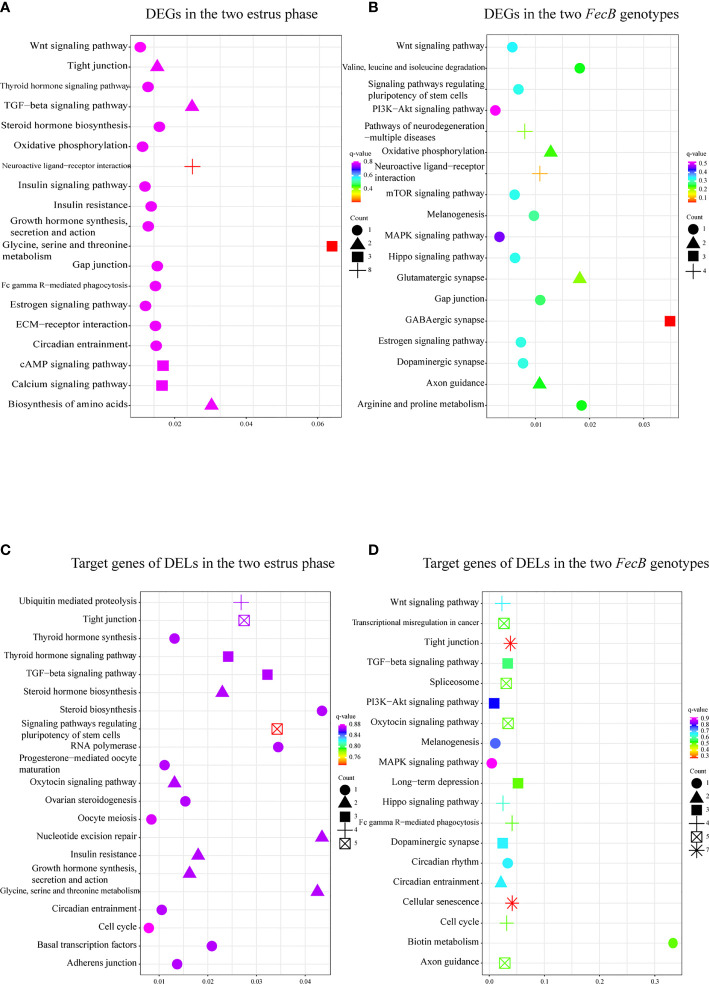
Enriched Kyoto Encyclopedia of Genes and Genomes (KEGG) pathways related to pituitary function and reproductive process of the differentially expressed genes (DEGs) and the target genes of differentially expressed lncRNAs (DELs). **(A, C)** KEGG enrichment pathways for DEGs **(A)** and the target genes of DELs **(C)** in the follicular phase and the luteal phase. **(B, D)** KEGG enrichment pathways for DEGs **(B)** and the target genes of DELs **(D)** in the BB (*FecB*-mutant homozygous) and WW (*FecB* wild type) genotypes.

The described methods were employed to predict the functions of the DEGs; that is, the enriched GO functional terms and KEGG pathways were considered to be the predicted functional terms and pathways for the protein-coding gene and lncRNA (47). In the comparison of the follicular and luteal phases, GO analysis of the DEGs showed that prolactin secretion, regulation of mitotic cell cycle, and amino acid metabolism were among the top 50 enriched terms (**Supplementary Table S3**). In addition, 125 pathways were annotated to hormone synthesis, secretion, and action in follicular–luteal transition, including steroid hormone, growth hormone, thyroid hormone, estrogen, and insulin (**Figure 3A** and **Supplementary Table S3**).

DEGs between the different *FecB* genotypes were enriched in pituitary hormone signal transduction processes (in the top 50 enriched terms), such as neurotransmitter metabolic process, choline metabolic process, and regulation of synapse process (**Supplementary Table S3**). KEGG pathway analysis identified 65 enriched pathways, including neuroactive ligand–receptor interaction, oxidative phosphorylation, dopaminergic synapse, melanogenesis, gap function, Wnt, mTOR, MAPK, PI3K–Akt, and Hippo signaling pathways (**Figure 3B** and **Supplementary Table S3**).

### Screening of Potential Functional LncRNAs Involved in Small Tail Han Sheep Reproduction

**Figure 4:**
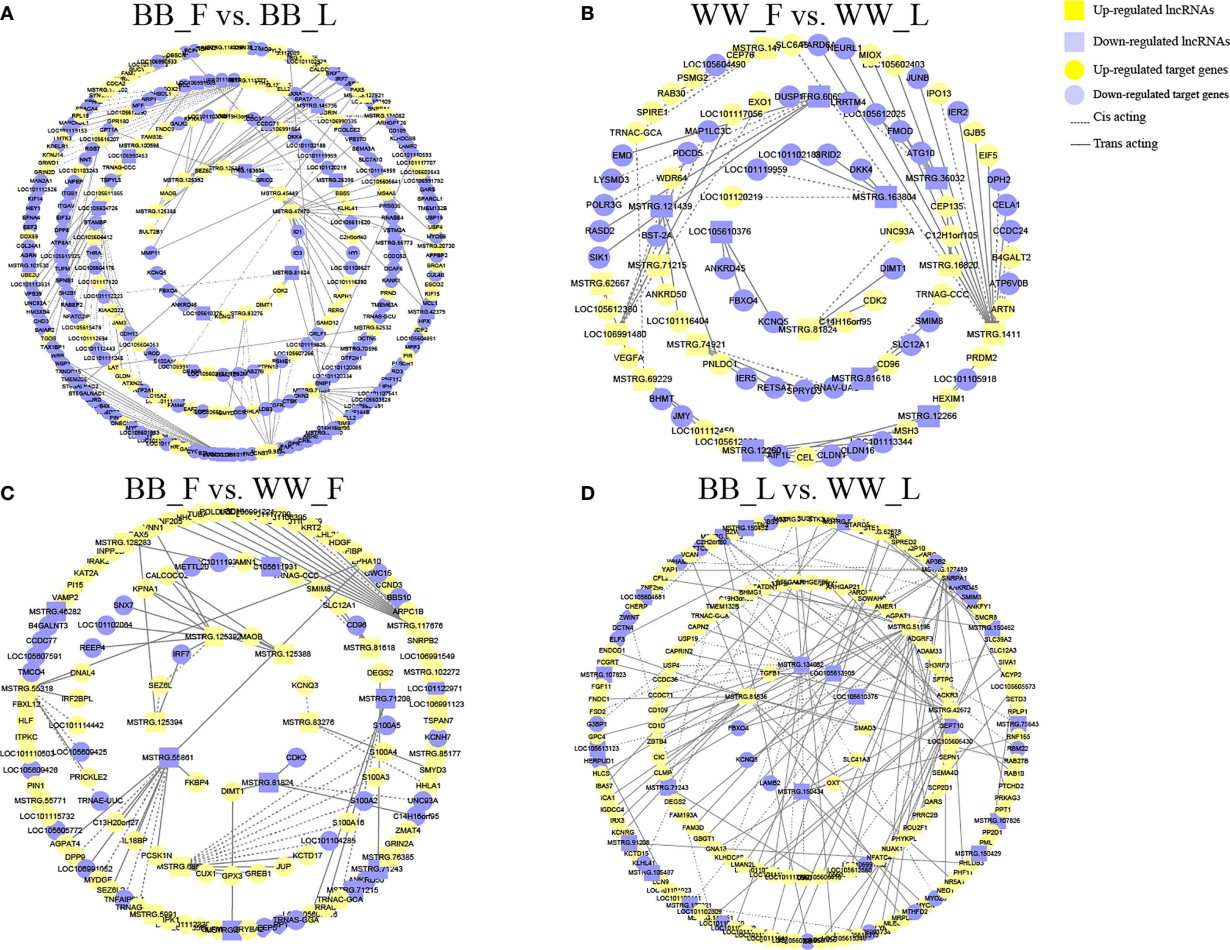
Interactions of the differentially expressed lncRNAs (DELs) with target genes form a network. **(A)** In the comparison BB_F *vs*. BB_L, 40 DELs acting in *cis* or in *trans* with 275 messenger RNAs (mRNAs) formed the interactive network. *Green* and *red colors* denote upregulated and downregulated long non-coding RNAs (lncRNAs), respectively. *Quadrilaterals* and *boxes* denote lncRNAs and target genes, respectively. *Dotted* and *straight lines* represent *cis*-acting and *trans*-acting, respectively. **(B)** In the comparison WW_F *vs*. WW_L, 17 DELs acting in *cis* or in *trans* with 83 mRNAs formed the interactive network. **(C)** In the comparison BB_F *vs*. WW_F, 23 DELs acting in *cis* or in *trans* with 117 mRNAs formed the interactive network. **(D)** In the comparison BB_L *vs*. WW_L, 27 DELs acting in *cis* or in *trans* with 151 mRNAs formed the interactive network. *F*, follicular phase; *L*, luteal phase; *BB*, *FecB*-mutant homozygous genotype; *WW*, *FecB* wild-type genotype.

To further reveal the potential biological function of pituitary lncRNAs in Small Tail Han sheep, an interaction network of lncRNAs and their corresponding target genes was constructed. In the comparison BB_F *vs*. BB_L, 12 known lncRNAs corresponded to 86 target genes, and 28 novel lncRNAs corresponded to 189 target genes (**Figure 4A** and **Supplementary Table S5**). In the comparison WW_F *vs*. WW_L, two known lncRNAs corresponded to eight target genes, and 15 novel lncRNAs corresponded to 75 target genes (**Figure 4B** and **Supplementary Table S5**). In the comparison of the follicular and luteal phases, the top 50 enriched terms of the target genes included tight junction, steroid hormone binding, and transcription elongation factor complex (**Supplementary Table S4**). In addition, 144 pathways were related to circadian entrainment, oocyte meiosis and maturation, and hormone signaling pathways, including thyroid hormone, oxytocin, and growth hormone (**Figure 3C** and **Supplementary Table S4**). These follicular–luteal transition DELs might play similar roles in pituitary hormone synthesis and secretion. In **Table 2**, four DEGs and six genes targeted by DELs are shown in detail. The targets of lncRNAs MSTRG.125392, MSTRG.125394, and MSTRG.83276 were *cis*-regulated, while others were *trans*-regulated. The expression patterns of most lncRNAs were similar to those of their corresponding target genes during the follicular–luteal transition.

**Table 2:** Details of the differentially expressed genes (DEGs) and genes targeted by differentially expressed lncRNAs (DELs).

Group	Co-expressed DE lncRNA	Chromosome location	Regulation	Interaction	Target gene	Chromosome location	Significant	Regulation	Main pathway enrichment
Gene set 1	MSTRG.47470	chrNC_019461.2:456882–459967:+	Up	*Trans*	*ID1*	chrNC_019470.2:60371052–60389936:+	No	Down	TGF-beta signaling
					*ID3*	chrNC_019459.2:242182555–242184262:+	No	Down	TGF-beta signaling
	MSTRG.101530	chrNC_019469.2:77360829–77371427:−	Down	*Trans*	*EEF2*	chrNC_019462.2:17534898–17543680:+	Yes	Down	Oxytocin signaling
	MSTRG.71854	chrNC_019464.2:40913326–40965345:+	Up	*Trans*	*LOC101107541*	chrNC_019470.2:42409305–42423098:+	No	Down	Steroid hormone biosynthesis, ovarian steroidogenesis
	MSTRG.97510	chrNC_019469.2:12548798–12553926:+	Down	*Trans*	*CREB5*	chrNC_019461.2:67260581–67700539:−	No	Down	Growth hormone synthesis, secretion and action, dopaminergic synapse, thyroid hormone synthesis, and estrogen signaling
Gene set 2	MSTRG.55861	chrNC_019462.2:16987989–16994238:−	Down	*Cis*	*FKBP4*	chrNC_019460.2:210696383–210703749:−	No	Up	Estrogen signaling
Gene set 3	LOC105613905	chrNC_019471.2:3212625–3218508:−	Down	*Trans*	*TGFB1*	chrNC_019471.2:49553525–49567792:+	No	Up	TGF-beta signaling pathway, cell cycle, and MAPK signaling
	MSTRG.81536	chrNC_019465.2:82316174–82350430:−	Up	*Trans*	*SMAD3*	chrNC_019464.2:13767248–13795543:+	No	Up	Cell cycle, TGF-beta signaling, Wnt signaling, and adherens junction
	MSTRG.150434	chrNC_019481.2:14769241–14775628:+	Down	*Trans*	*OXT*	chrNC_019470.2:51351726–51352626:−	No	Up	Oxytocin signaling, cAMP signaling, and neuroactive ligand–receptor interaction
Gene set 5	MSTRG.81824	chrNC_019465.2:88605603–88610913:−	Down	*Trans*	*CDK2*	chrNC_019460.2:163059743–163065314:−	No	Up	Progesterone-mediated oocyte maturation, oocyte meiosis, and cell cycle
			Down					Down	
Gene set 6	MSTRG.125392	chrNC_019474.2:65634940–65648893:+	Up	*Cis*	*SEZ6L*	chrNC_019474.2:65695000–65762617:+	Yes	Up	
	MSTRG.125394	chrNC_019474.2:65650268–65652112:+	Up	*Cis*					
	MSTRG.83276	chrNC_019466.2:21951292–21953221:+	Up	*Cis*	*KCNQ3*	chrNC_019466.2:21654282–21947569:+	Yes	Up	

In the comparison BB_F *vs*. WW_F, two known lncRNAs corresponded to four target genes, and 21 novel lncRNAs corresponded to 113 target genes (**Figure 4C** and **Supplementary Table S5**). In the comparison BB_L *vs*. WW_L, six known lncRNAs corresponded to 19 target genes, and 21 novel lncRNAs corresponded to 132 target genes (**Figure 4D** and **Supplementary Table S5**). In the comparison of the *FecB* genotypes, adenosine receptor binding, postsynaptic cytosol, kinase activity, and neurohypophyseal hormone activity were enriched in the top 50 terms (**Supplementary Table S4**). In addition, 208 pathways were identified, which included tight junction, TGF-beta, Hippo, Wnt, circadian rhythm, melanogenesis, MAPK, and PI3K–Akt signaling pathways (**Figure 3D** and **Supplementary Table S4**). Enrichment analyses of the DELs and DEGs showed similarities, indicating that DELs might be involved in the process by which *FecB* mutation had an effect on pituitary function. Two DEGs and five genes targeted by DELs are shown in detail (**Table 2**).

### Validation of RNA Sequencing Using RT-qPCR

**Figure 5:**
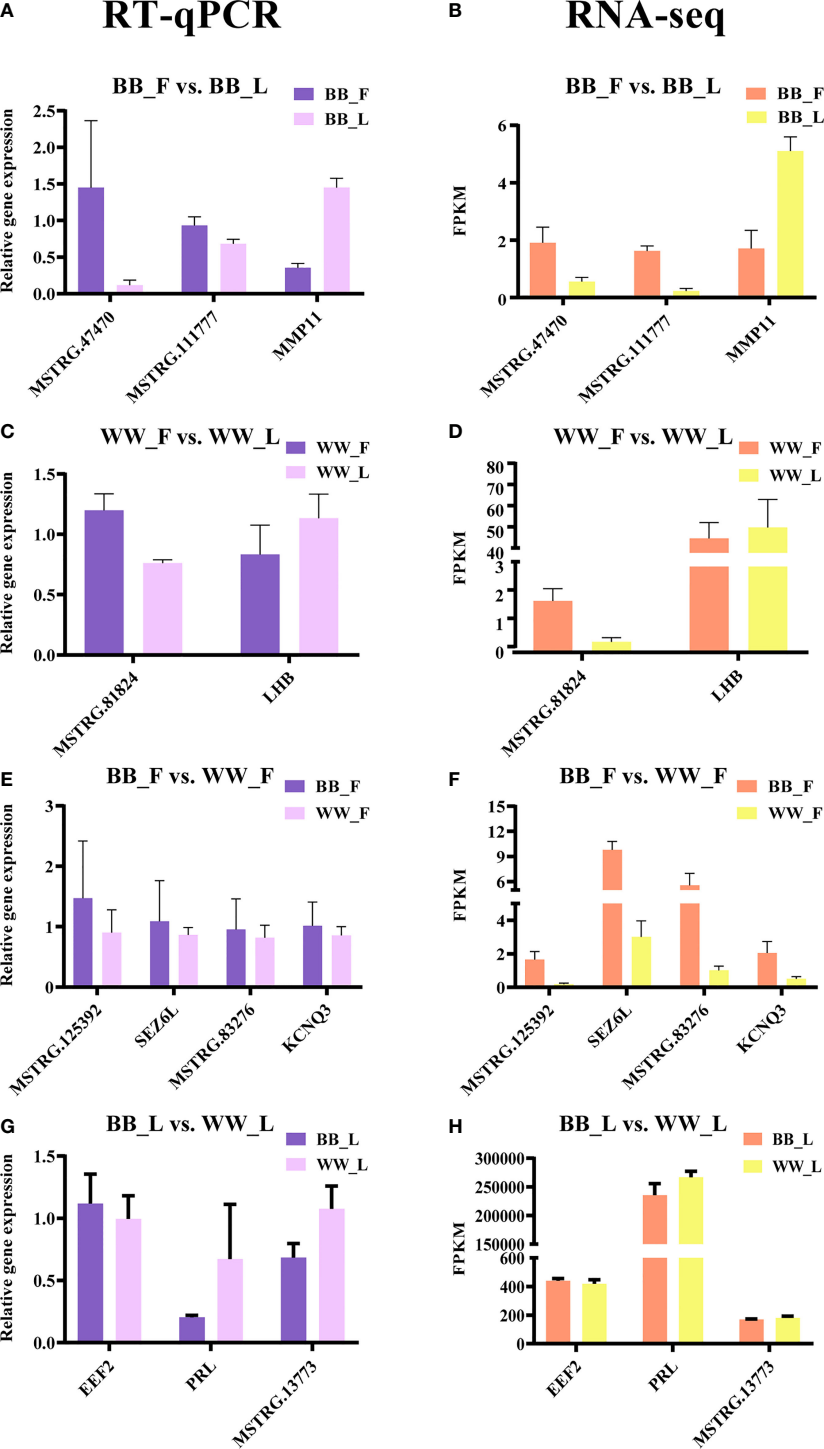
Validation of the RNA sequencing (RNA-seq) data by real-time quantitative PCR (RT-qPCR). The RT-qPCR data are presented as relative gene expression. RNA-seq data are presented as fragments per kilobase of transcripts per million mapped reads (FPKM). **(A–D)** Selected genes and long non-coding RNAs (lncRNAs) from BF and BL **(A, B)** and from WF and WL **(C, D)** were validated by RT-qPCR and RNA-seq, respectively. **(E–H)** Selected genes and lncRNAs from BF and WF **(E, F)** and from BL and WL **(G, H)** were validated by RT-qPCR and RNA-seq, respectively. BF, FecB-mutant homozygous genotype ewes in the follicular phase; BL, FecB-mutant homozygous genotype ewes in the luteal phase; WF, FecB wild-type genotype ewes in the follicular phase; WL, FecB wild-type genotype ewes in the luteal phase.

To validate the RNA-seq data, eight genes and eight lncRNAs were examined with RT-qPCR, and the relative gene expression was calculated on the basis of the 2^−ΔΔCt^ values (**Figure 5**). The results indicated that the data on the mRNA and lncRNA expression levels were reliable. Meanwhile, the results of Pearson’s correlation analysis of all genes showed a strong positive correlation between the RT-qPCR and RNA-seq data (*r* ^2^ > 0.93, *p* < 0.05).

## Discussion

Pituitary gonadotropin is essential for the activation of reproduction through the synthesis and secretion of gonadotropic hormones (FSH and LH) at the required levels. Given the unique function of the pituitary, i.e., the production of FSH and LH at specific times and appropriate levels, and its response to diverse hormone signals, it is expected that some regulatory elements play dominant roles in this cell-specific expression and hormonal responsiveness. In our study, 8,562 novel lncRNAs identified from the sheep pituitary shared many characteristics with those from other mammals, such as goats, mice, and pigs. In these mammals, most lncRNAs possessed two exons (followed by three exons), and lncRNAs were shorter and lower than mRNAs and were expressed at lower levels, reinforcing the legitimacy of the transcripts identified here (48, 49). Fewer lncRNAs were identified here than in the hypothalamus and pituitary gland of sheep, which had 34,293 and 19,672 lncRNAs, respectively (25, 50), likely reflecting tissue-specific expression patterns and characteristics and the different sampling times employed. The most lncRNAs in the present study were detected on ovine chromosomes 1–3, which was perhaps unsurprising as these chromosomes are the largest in the ovine genome. This implies a relationship between these chromosomes and pituitary function in the context of the *FecB* mutant. Similar chromosome locations of lncRNAs were found in previous studies on Hu sheep (chromosomes 1, 2, and 3) (25). In other words, the total distribution of lncRNAs was consistent between Hu sheep and Small Tail Han sheep, indicating that the functions of these lncRNAs might be conserved. Meanwhile, 20,248 mRNAs were identified, including *OXTR*, which is associated with the neurohypophysis, and hormone genes (*FSHB*, *LHB*, *PRL*, and *INHA*) and receptors (*FSHR*, *ESR1*, and *ESR2*) associated with the adenohypophysis (51, 52).

To understand the regulatory mechanism of the follicular–luteal transition, a previous study focused mainly on the microRNA (miRNA)–mRNA interactions in ovarian tissue and revealed that some miRNAs, such as miR-200a, miR-200b, and miR-200c, appeared to be critical regulators of ovarian follicular and luteal development (53, 54). However, pituitary hormones (FSH and LH) are required for the transition from the follicle to the corpus luteum, which involves receiving feedback signals from gonadal steroids and activin/inhibin signaling pathways (55, 56). In our study, DEGs from the follicular–luteal transition of wild-type ewes were mainly enriched in the G2/M transition of mitotic cell cycle, response to BMP, and Wnt signaling pathway of the GO terms and tight junction, TGF-beta signaling pathway, circadian entrainment, gap junction, and hormone-related signaling pathways of the KEGG pathways. *IGFBP3*, *PLCE1*, and *GRM1* were annotated to hormone-related signaling pathways, which might belong to the adenohypophysis. Moreover, *GRM1* was annotated as a gap junction gene; gap junctions mediate intercellular communication or cytosolic connections to regulate the amount of gonadotropin produced (57). With the *FecB* mutation, the number of DEGs increased and were annotated to amino acid metabolic process, prolactin secretion, and melanin biosynthetic process of the GO terms and the neuroactive ligand–receptor interaction, amino acid metabolism, hormone biosynthesis, and synapse-related pathways of the KEGG pathways. *RLN3*, *GRM8*, *GRID2*, and *VIP* were enriched in neuroactive ligand–receptor interaction, and *GRM8*, *KCNQ3*, and *MAOB* were enriched in synapse-related pathways, suggesting that these genes might be part of the neurohypophysis. Among these DEGs, *GRID2* and *ST14* were downregulated in the follicular phase compared with the luteal phase. A previous study revealed that *GRID2* is the receptor for glutamate that controls anterior pituitary hormone secretion and reproduction (58, 59); that is, *GRID2* may promote LH secretion to be involved in ovulation like glutamate receptor AMPA 1 (60). *ST14* reduced cell growth by downregulating the cell cycle-related proteins, and *ST14* was detected in the pituitary, indicating that it played an important role in pituitary development (61). Meanwhile, we identified some RAB proteins, among which RAB1A and RAB26 had higher relative expressions. RAB proteins are master regulators of vesicle transport, which is an essential process for secretion (62). Reductions in RAB1B, RAB3B, RAB6, and RAB11 may disrupt pituitary secretion of FSH and LH (63). The current results suggested that RAB1A and RAB26 might be new candidates involved in the secretory pathway in the pituitary gland. Moreover, some regulatory elements are expected to play dominant roles in follicular–luteal transition. The lncRNA MSTRG.81824 was upregulated in the comparison of WW_F with WW_L, but downregulated in the comparison of BB_F with BB_L, indicating that *FecB* could decrease the expression level of MSTRG.81824 in the follicular phase. MSTRG.81824 may have a *trans* effect on *CDK2* (cyclin dependent kinase 2) and affect follicle maturation (53, 64, 65). Other lncRNAs (MSTRG.47470 and MSTRG.101530) worked in *trans* when they affected the target genes *ID1* and *ID3* (a DEG), and *EEF2*, respectively. *ID1* and *ID3*, and *EEF2* in the pituitary induced melatonin and peptide hormone secretion, indicating that MSTRG.47470 and MSTRG.101530 are associated with hormone secretion (66, 67).

Mulsant et al. reported that Small Tail Han sheep were characterized by a high ovulation rate and litter size due to the action of the major gene *FecB* (2). It was suggested that, in ewes with the BB genotype, the *BMPR1B* mutation would advance granulosa cell differentiation and ovulatory follicle maturation, resulting in differential gene expression in the pituitary gland (68). In our study, the *BMPR1B* gene did not reach a significant difference in the different genotypes. However, estrogen functions in ovarian tissue and is involved in the process by which it downregulates the production and secretion of pituitary gonadotropin *via* negative feedback to the pituitary (69). Comparison of BB_F *vs*. WW_F showed that the *FecB* mutation significantly increased the expression of the *WNT11* (Wnt family member 11) gene in the follicular phase. The expression levels of the three DELs (MSTRG.125392, MSTRG.125394, and MSTRG.83276) and the two target genes (*SEZ6L* and *KCNQ3*) in the pituitary of BB genotype ewes were significantly higher than those of WW genotype ewes, and the expression trends of lncRNAs and their corresponding *cis*-target genes were consistent. Moreover, *cis*-regulatory lncRNAs showed enhancer-like activity and promoted the expression of neighboring genes. The DEG *SEZ6L* was *cis*-regulated by MSTRG.125392 and MSTRG.125394 and has been reported to influence dendritic morphology and synapse numbers (70). Previous conservation genomic analysis identified *SEZ6L* as a candidate gene associated with reproduction and system development in Black Slavonian pigs through the modulation of key oncogenic pathways such as Wnt (71, 72). Meanwhile, the DEG *KCNQ3*, a *cis*-regulatory element of MSTRG.83276, was important in the regulation of NPY/AgRP neuron excitability, which promoted hormone communication to maintain homeostasis. These results indicated that these three DELs might regulate the transcription of the target genes by promoting the enhancer and the promoter of the transcriptional machinery molecule, similar to the manner in which classical lncRNAs (HOTTIP and HOTAIRM1) promoted the expression of the adjacent target gene *HoxA* by *cis* regulation (73). *FKBP4*, an element that is *trans*-regulated by MSTRG.55861, is relevant to steroid hormone receptor binding and transportation and to reproductive and neurological diseases (74). Our study showed that *FKBP4* was somewhat differentially expressed and might induce steroid hormone synthesis in pituitary tissue. As an important paralog of this gene, *FKBP5* (FKBP prolyl isomerase 5) might produce more FSH in the hypothalamus tissue (75); that is, *FKBP4* and *FKBP5* are closely tied to hormone synthesis and secretion in the hypothalamic–pituitary–ovarian (HPO) axis, and *FecB* affects their expression trend. In the luteal phase in different *FecB* genotypes, the target genes *TGFB1* (transforming growth factor beta 1), *SMAD3* (SMAD family member 3), and *OXT* (oxytocin/neurophysin I prepropeptide) were *trans*-elements of the lncRNAs LOC105613905, MSTRG.81536, and MSTRG.150434, respectively. It was suggested that, with *FecB* mutation, lncRNAs in the follicular phase might be involved in pituitary development and steroid hormone communication, while lncRNAs in the luteal phase probably participate in the TGFβ/SMAD signaling pathway and in oxytocin secretion.

Overall, our results suggested that some genes were differentially expressed in the pituitary tissue between the follicular and luteal phases *via* the regulation of reproductive hormone synthesis and secretion. During the follicular phase, the expression of some genes in the pituitary tissue was elevated in the *FecB* mutant in comparison with the wild-type genotype. Whether the change in the gene expression levels affected the corresponding reproductive pathways and processes remains to be further elucidated.

## Conclusions

This pituitary transcriptome analysis showed the expression profiles of the pituitary tissue with the *FecB* mutation in the follicular–luteal transition. The comparison of the follicular phase and the luteal phase suggested that lncRNAs might be implicated in hormone secretion. In ewes with the *FecB* mutation, lncRNAs in the follicular phase (MSTRG.125392, MSTRG.125394, MSTRG.83276, and MSTRG.55861) might be associated with pituitary development and steroid hormone communication, while those in the luteal phase (LOC105613905, MSTRG.81536, and MSTRG.150434) probably participated in the TGFβ/SMAD signaling pathway and in oxytocin secretion. Moreover, the data obtained in this study should be useful for improving breed conservation and for better exploiting the genetic resources of sheep.

## Data Availability Statement

The datasets presented in this study can be found in online repositories. The names of the repository/repositories and accession number(s) can be found below: https://www.ncbi.nlm.nih.gov/, accession ID: PRJNA782215.

## Ethics Statement

All experimental ewes were supported by the Science Research Department of the Institute of Animal Sciences, Chinese Academy of Agriculture Sciences (IAS-CAAS). Ethical approval was in compliance with the Animal Ethics Committee of the IAS-CAAS (no. IAS 2019-49).

## Author Contributions

XG, XW, and MC conceived and designed the experiments. XG and XW performed the experiments. SC analyzed the data and wrote the manuscript, with input from the other authors. All authors contributed to the article and approved the submitted version.

## Funding

This research was funded by The National Natural Science Foundation of China (32172704 and 31902150), the Earmarked Fund for China Agriculture Research System of MOF and MARA (CARS-38), the Central Public-Interest Scientific Institution Basal Research Fund (Y2017JC24 and 2018ywf-yb-2), the Agricultural Science and Technology Innovation Program of China (CAAS-ZDRW202106 and ASTIP-IAS13), the Natural Science Foundation of Tianjin (20JCQNJC00630), the Natural Science Foundation of Jilin Province (20210101376JC), and China Postdoctoral Science Foundation (2021M703202).

## Conflict of Interest

The authors declare that the research was conducted in the absence of any commercial or financial relationships that could be construed as a potential conflict of interest.

## Publisher’s Note

All claims expressed in this article are solely those of the authors and do not necessarily represent those of their affiliated organizations, or those of the publisher, the editors and the reviewers. Any product that may be evaluated in this article, or claim that may be made by its manufacturer, is not guaranteed or endorsed by the publisher.

## Supplementary Material

Supplementary Table S1 Alignment of statistical results of reads.

Supplementary Table S2 The results of differentially expressed mRNAs and lncRNAs.

Supplementary Table S3 DEGs GO and KEGG enrichment analysis.

Supplementary Table S4 Target genes of DELs GO and KEGG enrichment analysis.

Supplementary Table S5 The lncRNAs-mRNAs network

Supplementary Table S6 Primer sequence and product size of mRNAs and lncRNAs for RT-qPCR.

Supplementary Figure S1 The Pearson correlation between samples.
