# Supplementary figures and images for: Insight Into Pituitary lncRNA and mRNA at Two Estrous Stages in Small Tail Han Sheep With Different FecB Genotypes
Source: Front Endocrinol (Lausanne). 2022 Feb 1;12:789564. doi: 10.3389/fendo.2021.789564 (PMC8844552; doi:10.3389/fendo.2021.789564)

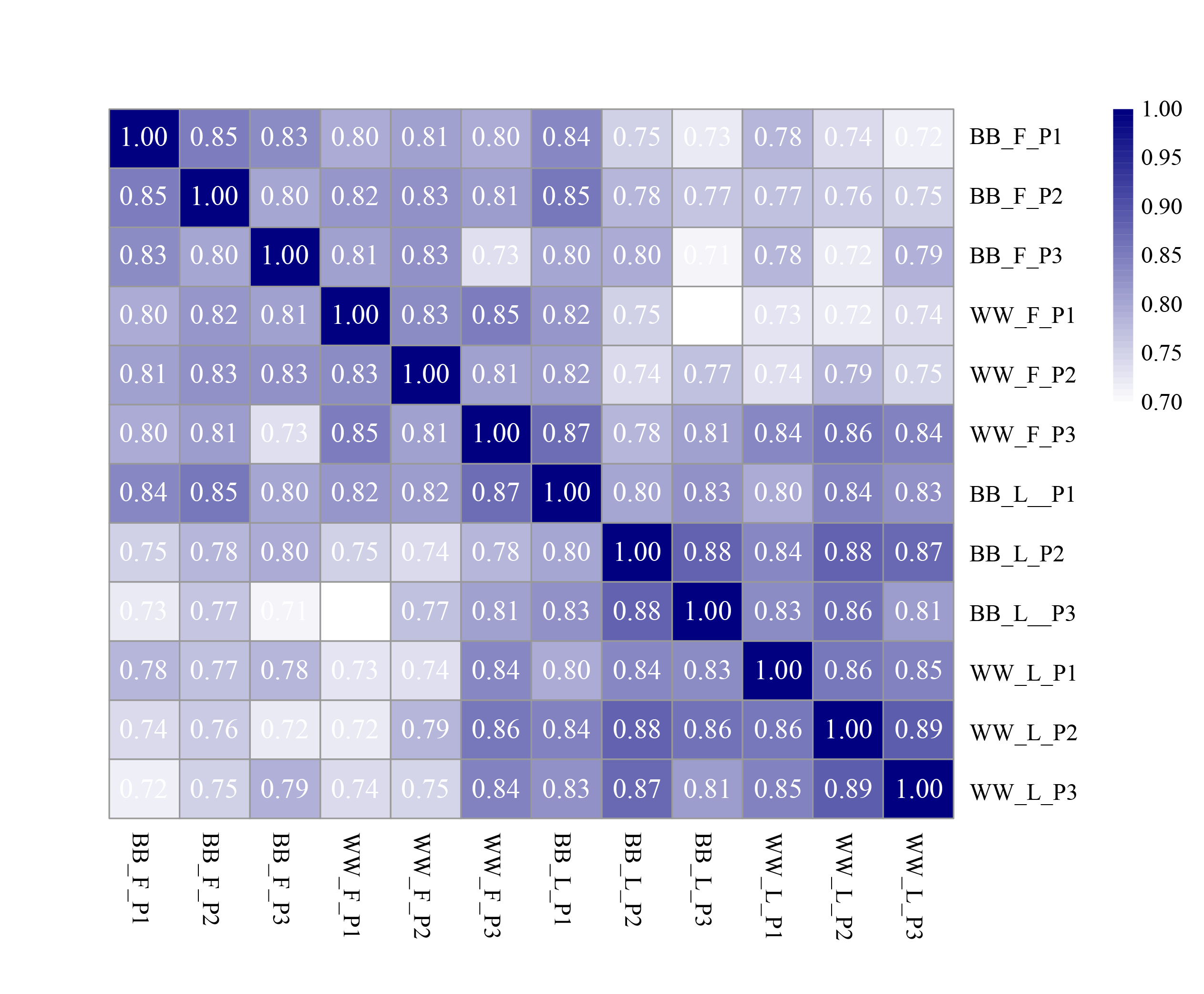

Supplement: Supplementary Figure S1 — The Pearson correlation between samples. [file Image_1.tif]
